# Amplicon-Based High-Throughput Sequencing Method Capable of Species-Level Identification of Coagulase-Negative Staphylococci in Diverse Communities

**DOI:** 10.3390/microorganisms8060897

**Published:** 2020-06-14

**Authors:** Emiel Van Reckem, Luc De Vuyst, Frédéric Leroy, Stefan Weckx

**Affiliations:** Research Group of Industrial Microbiology and Food Biotechnology (IMDO), Faculty of Sciences and Bio-engineering Sciences, Vrije Universiteit Brussel, B-1050 Brussels, Belgium; Emiel.Van.Reckem@vub.be (E.V.R.); luc.de.vuyst@vub.be (L.D.V.); frederic.leroy@vub.be (F.L.)

**Keywords:** coagulase-negative staphylococci, high-throughput sequencing, metagenetics

## Abstract

Coagulase-negative staphylococci (CNS) make up a diverse bacterial group, appearing in a myriad of ecosystems. To unravel the composition of staphylococcal communities in these microbial ecosystems, a reliable species-level identification is crucial. The present study aimed to design a primer set for high-throughput amplicon sequencing, amplifying a region of the *tuf* gene with enough discriminatory power to distinguish different CNS species. Based on 2566 *tuf* gene sequences present in the public European Nucleotide Archive database and saved as a custom *tuf* gene database in-house, three different primer sets were designed, which were able to amplify a specific region of the *tuf* gene for 36 strains of 18 different CNS species. In silico analysis revealed that species-level identification of closely related species was only reliable if a 100% identity cut-off was applied for matches between the amplicon sequence variants and the custom *tuf* gene database. From the three primer sets designed, one set (Tuf387/765) outperformed the two other primer sets for studying *Staphylococcus*-rich microbial communities using amplicon sequencing, as it resulted in no false positives and precise species-level identification. The method developed offers interesting potential for a rapid and robust analysis of complex staphylococcal communities in a variety of microbial ecosystems.

## 1. Introduction

*Staphylococcus* is a genus of Gram-positive, catalase-positive, facultative anaerobic bacteria that belongs to the family Staphylococcaceae, order Bacillales [1]. This genus currently contains 53 species and 28 subspecies (www.bacterio.net; Leibniz Institute DSMZ, 2019). Historically, the coagulase test was used to make a distinction between the pathogenic *Staphylococcus aureus*, which is a coagulase-positive staphylococcal species, and less or non-pathogenic staphylococci, which were considered to be coagulase-negative. The latter group was therefore referred to as coagulase-negative staphylococci (CNS) [2]. Alternatively, the term non-aureus staphylococci (NAS) has been proposed, as several studies have revealed, that some *Staphylococcus* spp. show a variable response to the coagulase test, such as *Staphylococcus agnetis* and *Staphylococcus chromogenes* [3,4,5]. CNS or NAS form a very large and genetically diverse phylogenetic group, consisting of five distinct clades based on whole genome sequencing analysis, spanning a wide variety of microbial ecosystems [5]. As such, they are known not only as opportunistic pathogens in humans and animals, for example as the main cause of intramammary infections, but also as valuable microorganisms for the production of fermented foods, such as fermented sausages [5,6,7]. *Staphylococcus equorum, Staphylococcus saprophyticus*, and *Staphylococcus xylosus* are most prominent CNS species in food fermentations, whereas species such as *S. chromogenes, Staphylococcus epidermidis, Staphylococcus haemolyticus, Staphylococcus hominis, S. saprophyticus*, and *Staphylococcus simulans* are most frequently encountered as pathogens in both humans and animals [8,9,10,11].

Given the presence of pathogenic species within the *Staphylococcus* genus and hence the importance of a correct identification, a wide array of approaches has been applied for culture-dependent species-level identification. In general, genotypic methods, such as (GTG)_5_-PCR fingerprinting of genomic DNA, are considered superior compared to phenotypic methods, as the latter are often less sensitive and prone to misidentifications, primarily due to possible intra-species phenotypic variations [7,12,13]. Additionally, matrix-assisted laser desorption/ionization time-of-flight mass spectrometry (MALDI-TOF MS) of cell extracts is increasingly used for dereplication and identification of isolates, for which there is no needs for genomic DNA extraction [7]. Culture-independent identification methods, such as denaturing gradient gel electrophoresis of PCR amplicons (PCR-DGGE) targeting specific genomic regions, have been extensively used to study CNS isolates and bacterial communities containing CNS, respectively [14,15,16,17]. Culture-independent approaches offer several advantages, as they are generally not biased toward specific microorganisms and can also detect less abundant species [17].

Recently, high-throughput sequencing (HTS) technologies have been applied to study the totality of bacterial communities of an ecosystem, frequently by sequencing one or more hypervariable region(s) of their 16S rRNA genes [18,19,20]. However, the 16S rRNA gene has little discriminatory power within the *Staphylococcus* genus [21,22]. To circumvent this, it is necessary to use other phylogenetic marker genes that have a sufficient level of discriminatory power, such as the *gap*, *hsp60*, or *tuf* genes, when studying complex communities of staphylococcal species [22,23]. Previously, a method has been developed for species-level analysis of staphylococci in the nasal microbiome based on partial sequences of the *rpoB* gene [24]. Similarly, a previous study applying primers targeting the *tuf* gene and using only clinical isolates has shown that partial *tuf* gene amplicon sequencing can be used to unravel staphylococcal communities in the nasal microbiome [23]. Furthermore, the *tuf* gene has also been used successfully for the identification of CNS species present on the teat apices of dairy cows, albeit using PCR-DGGE [25].

The aim of the present study was to develop and validate a generic amplicon-based HTS method targeting the *tuf* gene for analysing a broad spectrum of staphylococcal communities, disregarding the ecosystem origin of the staphylococcal strains and species.

## 2. Materials and Methods

### 2.1. Bacterial Strains

To construct mock communities containing staphylococcal DNA to evaluate the culture-independent identification method developed in the current study, 36 bacterial strains were used, belonging to 18 different CNS species, namely *Staphylococcus auricularis, Staphylococcus arlettae, Staphylococcus carnosus, S. chromogenes, Staphylococcus cohnii, S. epidermidis, S. equorum, Staphylococcus fleuretti, S. hominis, S. haemolyticus, Staphylococcus lugdunensis, Staphylococcus pasteuri, S. saprophyticus, Staphylococcus sciuri, S. simulans, Staphylococcus succinus, Staphylococcus warneri*, and *S. xylosus* (Table 1). Species were selected to fully represent the genetic diversity present within the *Staphylococcus* genus, i.e., all five clades previously established using whole genome sequencing-based phylogenetic analysis are represented in the current study [5]. Per CNS species, two strains coming from the laboratory collection available at the research group IMDO were selected, transferred from −80 °C stocks to brain heart infusion (BHI) medium (Oxoid, Basingstoke, Hampshire, UK), and incubated overnight at 30 °C to acquire grown cultures to be used for DNA extraction.

Furthermore, the strains *S. carnosus* IMDO-S15, *S. epidermidis* IMDO-S30, *S. equorum* IMDO-S35, *S. saprophyticus* IMDO-S59, and *S. xylosus* IMDO-S81 were additionally grown in BHI medium and incubated at 30 °C for 12 h. At two different time points, namely 4 and 12 h, samples of these cultures were taken and pooled to create three cell-based mock communities to assess the discriminatory power of the culture-independent identification method developed, namely one aiming to have all species equally represented and two aiming to represent the species in a staggered distribution, meaning that some species were represented by much more cells than other species (see Section 3.1). To determine the colony forming units per mL after 4 and 12 h in a culture-dependent way, information that can serve as a guideline for the expected relative abundance of each CNS species present in the two types of mock communities used, serial decimal dilutions of the aforementioned cultures were prepared in a sterile saline solution, which were plated on mannitol salt phenol-red agar (MSA) medium (VWR International, Darmstadt, Germany), a medium that is suitable for both growth and recovery of CNS [26], and incubated at 30 °C for 72 h for bacterial enumeration. Although several other media exist for enumeration of staphylococci, including Mueller-Hilton agar or Tryptone soya agar (TSA) that are frequently used in medical microbiological laboratories, the choice of the medium to culture strains for mock community construction is not related to (and neither influences) the performance of the culture-independent identification method developed in the current study.

### 2.2. Genomic DNA Extraction

#### 2.2.1. Pure Cultures

Genomic DNA extraction from cell pellets, procured by microcentrifugation at 13,000 rpm of 1.5 mL of an overnight culture of the strains mentioned above, was performed with a Nucleospin 96 tissue kit (Macherey Nagel, Düren, Germany), according to the manufacturer’s instructions. Ahead of extraction, all cell pellets were washed with Tris-ethylenediaminetetraacetic acid (EDTA)-sucrose buffer [TES buffer; 50 mM Tris base (Calbiochem, Darmstadt, Germany), 1 mM EDTA (Sigma-Aldrich, St. Louis, MO, USA), and 6.7% (*m*/*v*) sucrose (VWR International); pH 8.0].

#### 2.2.2. Pooled Cultures

To obtain DNA of the cell-based mock communities as described in 2.1, aiming to account for differences in cell lysis efficiency, cell pellets were obtained from the pooled cultures by centrifugation at 4000× *g* at 4 °C for 20 min. Subsequently, DNA extraction was performed combining enzymatic, chemical, and mechanical cell lysis, followed by phenol/chloroform/isoamylalcohol extraction and column purification, as described previously [27]. However, since the pooled cultures did not contain any moulds or yeasts, the enzymatic steps to lyse them, using lyticase and Longlife Zymolyase, were omitted from the original protocol.

### 2.3. Primer Design

To search for conserved regions that could be used as targets for primer design, a multiple sequence alignment of 2566 publicly available *tuf* gene sequences of all staphylococcal species present in the European Nucleotide Archive of the European Bioinformatics Institute (ENA-EBI) database (Coding release; accessed April 2019) was performed using MUSCLE v3.8.31 [28]. From the resulting multiple sequence alignment, a consensus sequence (Table S1) was created using the EMBOSS package consambig [29]. Subsequently, three primer sets intended for high-throughput amplicon sequencing, further referred to as Tuf108/408, Tuf387/765, and Tuf216/522, wherein the numbers correspond to the position of the first nucleotide of the primer in the complete *tuf* gene, and one primer set amplifying a larger part of the *tuf* gene (Tuf32/900), were designed using Primer3 (Table 2) [30,31,32]. Since the multiple sequence alignment of the 2566 *tuf* gene sequences showed that the gene did not contain conserved regions long enough to design primer without degenerate positions, maximally three such degenerate positions were allowed per primer in the design process to accommodate this gene sequence variability. For most primers, such positions were needed to allow primer selection, although the majority of the primers had a degree of degeneracy of 8, with a maximum of 12, well below a degree of degeneracy of 128 that is considered the maximum allowable. The primer sets to be used for amplicon sequencing were extended with an Illumina platform-specific 5′ tag, as described previously [33]. To evaluate in silico the specificity of the primer sets designed, an online Primer-BLAST search was performed [National Center for Biotechnological Information (NCBI), Bethesda, MD, USA]. Hereto, the primer pairs were aligned to sequences belonging to the group of *Staphylococcaceae* present in the non-redundant RefSeq database and the non-redundant nucleotide database nt of the NCBI [34]

Furthermore, using the previously obtained multiple sequence alignment, the evolutionary divergence over sequence pairs was calculated by assessing the number of base differences within a species (intra-species) and between species (inter-species), using the sequences present in the custom *tuf* gene database and the software MEGA X [35]. For the intra-species differences, the number of base differences per sequence, averaged over all sequence pairs, within each species was calculated, whereas for the inter-species differences, the number of base differences per sequence, averaged over all sequence pairs, between a species and all other species was calculated. These numbers were further quantified as median, first quartile (Q1) and third quartile (Q3). The sequence differences were calculated to showcase the degree of conservation and variance of the *tuf* gene within the *Staphylococcus* genus and its suitability for typing.

### 2.4. PCR Assays and Sequencing

To optimize the actual annealing temperature, a gradient PCR was performed for all primer sets designed, using genomic DNA obtained from pure cultures of the above-mentioned 36 strains belonging to 18 different CNS species. The PCR assay conditions consisted of an initial denaturation step at 94 °C for 2 min, followed by 30 cycles of denaturation at 94 °C for 30 s, annealing with a gradient ranging from 54 to 64 °C for 60 s, and extension at 72 °C for 3 min, followed by a final extension at 72 °C for 7 min. For each PCR assay, the PCR mixture contained 5 μL of 10× PCR buffer (Roche Diagnostics, Mannheim, Germany), 0.2 mM of a deoxynucleotide triphosphate mixture (Sigma-Aldrich), 1.25 U of *Taq* DNA polymerase (Roche Diagnostics), 5 μM of each primer (Integrated DNA Technologies, Leuven, Belgium), and 10 ng of DNA [33].

**Figure 1:**
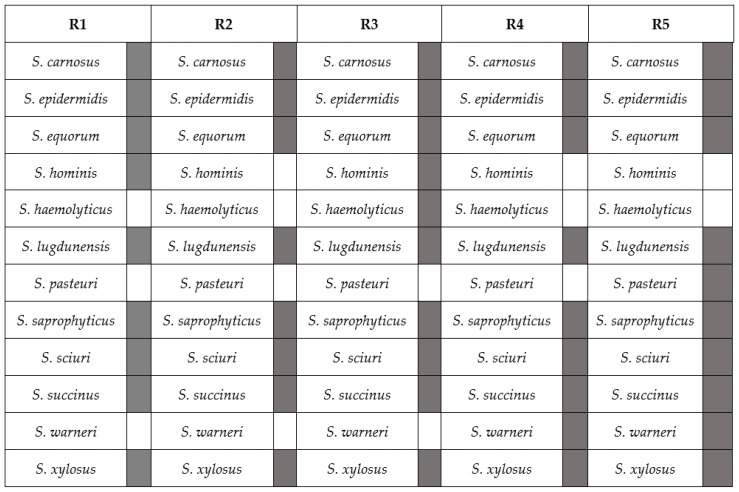
Staphylococcal species representation in different amplicon-based mock communities referred to as R1–R5. *S*. *staphylococcus*.

Using the optimized annealing temperature, genomic DNA from pure cultures of the 36 CNS strains was amplified using primer pair Tuf32/900. The PCR assay conditions consisted of an initial denaturation step at 94 °C for 2 min, followed by 30 cycles of denaturation at 94 °C for 30 s, annealing at 55 °C for 60 s, and extension at 72 °C for 3 min, followed by a final extension at 72 °C for 7 min. Subsequently, these 869-bp long amplicons were purified with a Wizard Plus SV gel and PCR clean-up system (Promega, Madison, WI, USA) and sequenced using the Sanger sequencing method (VIB Neuromics Support Facility, Antwerp, Belgium) to confirm their species identities. Hereto, the sequences obtained were aligned to the non-redundant nucleotide database nt of the NCBI, using BLAST as the alignment tool [36]. Next, from the sequences obtained, subsequences corresponding to the amplicons that would be obtained by using the Tuf108/408, Tuf387/765, and Tuf216/522 primer sets were extracted in silico. Those subsequences were aligned to the NCBI non-redundant nucleotide database nt, using BLAST as alignment tool for identification. The accession numbers matching these amplicons, as well as their percentage identities, are listed in Table S2. Further, based on measurements of their concentrations using a Qubit 2.0 fluorometer (Thermo Fisher Scientific, Waltham, MA, USA), the 869-bp amplicons were pooled to obtain amplicon-based mock communities, further denoted R1 to R5, with differing compositions of CNS species, in which the species were evenly represented (Figure 1). Whereas these amplicon-based mock communities R1 to R5 aim to assess the discriminatory power and relative abundances of the culture-independent identification method, developed in the current study based on the presence of a known amount of amplicons in the sample to be subjected to high-throughput sequencing and data analysis, the cell-based mock communities (see Section 3.1) have the same purpose, but here the variability due to differences in cell lysis efficiency, influencing the DNA extraction efficiency, is taken into account as well.

Finally, both the mock community containing DNA obtained from the pooled cultures as well as the amplicon-based mock communities were amplified using the Tuf108/408, Tuf387/765, and Tuf216/522 primer sets. The PCR assay conditions consisted of an initial denaturation step at 94 °C for 2 min, followed by 25 cycles of denaturation at 94 °C for 30 s, annealing at 55 °C for 60 s, and extension at 72 °C for 3 min, followed by a final extension at 72 °C for 7 min. The resulting amplicons were purified using a Wizard Plus SV gel and PCR clean-up system (Promega), and subjected to size selection using Agencourt AMPure XP PCR purification magnetic beads (Beckman Coulter, Brea, CA, USA). Subsequently, the PCR products were sequenced using an Illumina MiSeq sequencing platform (VUB-ULB BRIGHTcore sequencing facility, Jette, Belgium).

### 2.5. Amplicon Sequence Data Analysis

**Figure 2:**
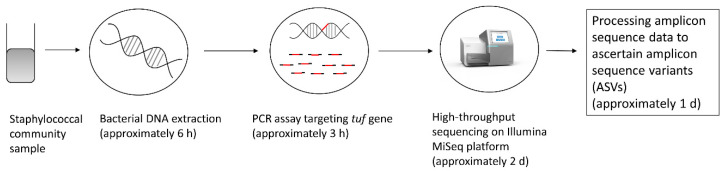
Overview of the workflow of the developed method, from staphylococcal community sample to obtaining ASVs, including an estimates of the time required for each step, as was the case for the present study (may vary substantially according to laboratory context).

Processing of the MiSeq sequence data was similar to the method described by Zhang et al. [37], whereby amplicon sequence variants (ASVs) were ascertained using the DADA2 package (version 1.10.1) within the open-source software environment R [38]. Taxonomy was assigned using a custom *tuf* gene database containing the above-mentioned 2566 *tuf* gene sequences of *Staphylococcus* species from the ENA/EBI database. Given the set-up of the current study, ASVs were only classified to species level if a 100% match with a sequence in the custom *tuf* gene database was found. Once species-level identification was achieved, the observed and expected relative abundances of the staphylococcal species in the different mock communities were compared by performing a chi-square test using the open-source software environment R. The complete workflow of the method developed, from staphylococcal community sample to obtaining ASVs, including an estimates of the time required for each step, is shown in Figure 2.

### 2.6. Data Availability

The amplicon sequencing data sets were submitted to the European Nucleotide Archive of the European Bioinformatics Institute (ENA/EBI) and are accessible under the study accession number PRJEB35705 (http://www.ebi.ac.uk/ena/data/view/PRJEB35705).

## 3. Results and Discussion

### 3.1. Enumeration and Pooling of Pure Cultures

The MSA counts of *S. carnosus* IMDO-S15, *S. epidermidis* IMDO-S30, *S. equorum* IMDO-S35, *S. saprophyticus* IMDO-S59, and *S. xylosus* IMDO-S81 amounted to 5.7, 5.3, 6.1, 6.9, and 6.2 log (CFU/mL) after 4 h of growth and to 8.2, 8.1, 8.1, 8.7, and 8.5 log (CFU/mL) after 12 h of growth, respectively. Appropriate volumes of the cultures were pooled after 12 h of growth to construct a cell-based, even mock community, aiming to obtain the same number of cells per species (further referred to as mock community M1). The resulting microbial loads in this mock community M1 were 7.5, 7.4, 7.4, 8.0, and 7.8 log (CFU/mL) for *S. carnosus* IMDO-S15, *S. epidermidis* IMDO-S30, *S. equorum* IMDO-S35, *S. saprophyticus* IMDO-S59, and *S. xylosus* IMDO-S81, respectively. Furthermore, a first staggered mock community was established, further referred to as mock community M2, in which S. *equorum* IMDO-S35, *S. saprophyticus* IMDO-S59, and *S. xylosus* IMDO-S81 were more abundant by combining cultures of the latter strains grown for 12 h with cultures of *S. carnosus* IMDO-S15 and *S. epidermidis* IMDO-S30 grown for 4 h. The resulting microbial loads in this mock community M2 were 5.0, 4.6, 7.4, 8.0, and 7.8 log (CFU/mL) for *S. carnosus* IMDO-S15, *S. epidermidis* IMDO-S30, *S. equorum* IMDO-S35, *S. saprophyticus* IMDO-S59, and *S. xylosus* IMDO-S81, respectively. Similarly, a second staggered mock community was established, further referred to as mock community M3, in which *S. carnosus* IMDO-S15 and *S. xylosus* IMDO-S81 were more abundant compared to the other staphylococcal species, with the microbial loads for *S. carnosus* IMDO-S15, *S. epidermidis* IMDO-S30, *S. equorum* IMDO-S35, *S. saprophyticus* IMDO-S59, and *S. xylosus* IMDO-S81 in this community reaching 7.5, 4.6, 5.4, 6.2, and 7.8 log (CFU/mL), respectively. All three mock communities were prepared in triplicate. Constructing both equal (M1) and staggered (M2 and M3) mock communities based on pooled cultures allowed for assessing the discriminatory power of the developed amplicon-based HTS method. Furthermore, enumeration of the pooled cultures allowed for calculating the expected relative abundance for each CNS species in the mock community. Since mock communities M1-M3 were also subjected to a DNA extraction, this allowed to assess the influence of the DNA extraction method on the diversity estimates found once correct species-level identification was achieved (Section 3.3).

### 3.2. Sequencing and In Silico Analysis

Reliable species-level identification of CNS is important to be able to unravel very diverse staphylococcal communities that occur in both clinical environments and (fermented) food systems [21,39]. Therefore, there was a need for a primer set to be used for amplicon sequencing, amplifying a region of the *tuf* gene with enough discriminatory power to distinguish different CNS species. To highlight the ability of the *tuf* gene to distinguish between different CNS species, the number of intra- (Table S3) and inter-species (Table S4) base differences were calculated using the sequences present in the custom *tuf* gene database. Differences between sequences related to the same staphylococcal species ranged from 0 to 13 nucleotides (nt) (median = 2 nt, Q1 = 0 nt, Q3 = 4 nt), whereas sequences from different staphylococcal species differed between 5 and 152 nt (median = 96 nt, Q1 = 82 nt, Q3 = 110 nt), thereby demonstrating the degree of intra-species conservation and inter-species variance of the *tuf* gene for species of the *Staphylococcus* genus, and showcasing its previously established discriminatory power between CNS species [5,21]. The latter is in accordance with previous studies showing that the *tuf* gene exhibits greater discriminatory power for staphylococcal species than the 16S rRNA gene [40,41]. Therefore, even though the 16S rRNA gene is currently the most targeted gene in amplicon-based HTS methods, the *tuf* gene appears to be a more appropriate target when studying diverse staphylococcal communities.

The PCR assays based on all four primer pairs designed showed amplification for all above-mentioned 36 strains belonging to 18 different CNS species. Furthermore, an additional in silico analysis using Primer-BLAST showed that primer pairs Tuf108/408, Tuf216/522, and Tuf387/765 were specific enough to align to all CNS species in the Refseq database and to multiple strains of these CNS species in the non-redundant nucleotide database nt, aligning with over 980 sequences across all CNS species, including prevalent species such as *S. capitis, S. epidermidis, S. equorum* and *S. xylosus*. Sequencing of the 869-bp amplicons and the following analysis, whereby subsequences were extracted in silico from the 869-bp sequences obtained, showed that the staphylococcal species *S. arlettae, S. chromogenes*, and *S. fleuretti* presumably could not be identified correctly (sequence identity <98%) based on the amplicons of primer pairs Tuf32/900, Tuf108/408, and Tuf216/522, whereas a correct CNS species assignment could be achieved with primer pair Tuf387/765 (Table S2). Furthermore, for *S. carnosus*, the amplicon that would be amplified by Tuf108/408 would not correctly identify the species, as the amplicon would be assigned to *S. condimenti*. Additionally, *S. haemolyticus* would not be correctly identified by any of the primer pairs Tuf32/900, Tuf108/408, or Tuf216/522, as it was identified as the closely related *S. hominis*. Therefore, in silico analysis showed that closely related CNS species, such as *S. haemolyticus* and *S. hominis*, could possibly be misidentified if the cut-off for species identification was set below 99% (Table S2). This corresponds with a previous study in which it was shown that some CNS species displayed a high sequence identity between one another, making correct identification more difficult [42]. While HTS technologies have greatly improved the capability to analyse complex microbial communities, an accurate and specific taxonomic classification is crucial [43]. Therefore, CNS species-level identification was only assigned if a 100% match of the ASV with a corresponding sequence in the custom *tuf* gene database was found. This information was also taken into account when constructing the amplicon-based mock communities, based on which closely related species, such as *S. haemolyticus* and *S. hominis*, were included to assess the CNS species resolution of the amplicon HTS method.

### 3.3. Amplicon-Based HTS Method

**Figure 3:**
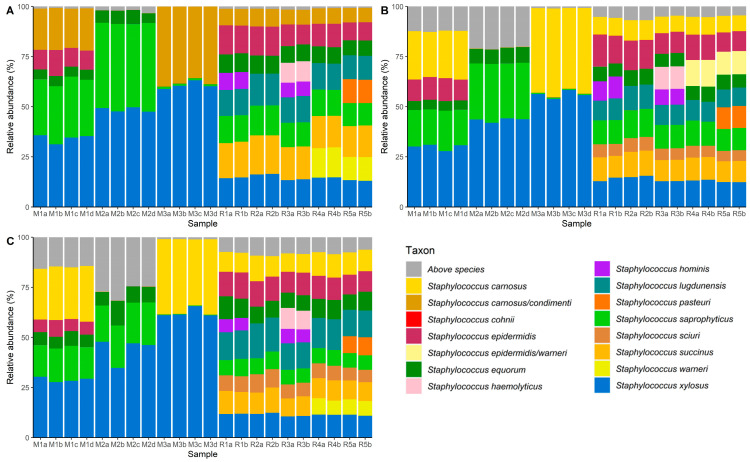
Distribution of amplicon sequence variants (ASVs) of the region of the *tuf gene* amplified by primer sets Tuf108/408 (**A**), Tuf216/522 (**B**), and Tuf387/765 (**C**) for cell-based mock communities M1, M2, and M3, and amplicon-based mock communities R1, R2, R3, R4, and R5. M1a, M1b, and M1c represent the three replicates of cell-based mock community M1, whereas M1d represents a PCR repetition using the same replicate as was used for M1a. Similar coding applies to cell-based mock communities M2 and M3. Analogously, R1a and R1b represent duplicates of the amplicon-based mock community R1. Similar coding applies for amplicon-based mock communities R2, R3, R4 and R5. Taxonomy was assigned to each ASV up to species level to assess the relative abundance of each species present in the mock communities. When unambiguous identification of staphylococcal species was not possible for a particular ASV, all species names corresponding with that ASV are mentioned.

The relative abundances of ASVs of the partial *tuf* gene, amplified by primer set Tuf108/408 for all mock communities, is displayed in Figure 3A.

Regarding the cell-based mock communities, *S. carnosus* could not be unambiguously identified by amplicon sequencing using Tuf108/408. The presumable *S. carnosus* ASV was instead assigned to both *S. carnosus* and *S. condimenti,* which have been previously reported as being closely related CNS species with a high degree of sequence identity and synteny [42,44]. In contrast, *S. epidermidis, S. equorum, S. saprophyticus*, and *S. xylosus* ASVs were correctly assigned and encountered in all cell-based mock communities. In mock community M2, wherein *S. carnosus* and *S. epidermidis* were less prevalent, and mock community M3, wherein *S. epidermidis, S. equorum*, and *S. saprophyticus* were less prevalent, the ASVs for the lesser abundant staphylococcal species were detected with a relative abundance higher than 0.01%. Regarding the amplicon-based mock communities, *S. carnosus, S. epidermidis, S. equorum, S. hominis, S. haemolyticus, S. lugdunensis, S. saprophyticus, S. succinus, S. xylosus, S. pasteuri*, and *S. warneri* were all correctly identified. However, *S. sciuri* was not found in any of the amplicon-based mock communities, although it was included. Therefore, primer set Tuf108/408 and the corresponding region of the *tuf* gene were not suitable for CNS species-level identification using amplicon sequencing.

Amplicon sequencing using primer set Tuf216/522 showed correct identification of all staphylococcal species, except for *S. warneri* (Figure 3B). The presumable *S. warneri* ASV was instead assigned to both *S. epidermidis* and *S. warneri.* In previous studies, both CNS species have been grouped in the same clade of a phylogenetic tree based on the *tuf* gene, high sequence identities being most likely again responsible for this misidentification [5,41]. Furthermore, in amplicon-based mock communities R1, R2, and R3, *S. cohnii* was found, albeit with a very low relative abundance (<0.02%), although in reality it was not included in the mock community. The latter was probably an artefact, resulting from amplification and sequencing, since it was only found in very low relative abundance [45,46]. Lesser abundant species in cell-based mock communities M2 and M3 could be distinguished with their relative abundances amounting to more than 0.01%.

In contrast to the above, all ASVs were correctly identified by amplicon sequencing using primer set Tuf387/765 in the cell-based mock communities as well as in the amplicon-based mock communities (Figure 3C). Lesser abundant species in cell-based mock communities M2 and M3 were still detected, with a relative abundance greater than 0.01%, and all species added in the amplicon-based mock communities were accurately identified. With more unassigned reads, due to stringent assignment criteria but yielding seemingly no false positives and precise species identification, Tuf387/765 was the best candidate primer set for studying staphylococci-rich microbial communities using high-throughput amplicon sequencing. Once correct species-level identification was achieved, the observed and expected relative abundances of the staphylococcal species in the different mock communities were compared (Table S5). Although the observed and expected relative abundances are relatively comparable for amplicon-based mock communities R1 to R5 (average difference of 2% in relative abundance), this was less the case for the cell-based mock communities M1 to M3 (average difference of 7% in relative abundance), for which the DNA extraction was performed on the pooled cell cultures. Nevertheless, observed and expected relative abundance were significantly correlated in both cases, for amplicon-based mock communities R1 to R5 (χ^2^ = 2640, *p* < 0.05), and the cell-based mock communities M1 to M3 (χ^2^ = 504, *p* < 0.05). However, despite the discriminatory power of the aforementioned method, care should be taken when interpreting diversity estimates, as similar to other PCR-based metabarcoding studies, several biases can be introduced that can slant these estimates [47]. However, due to its culture-independent nature, the method developed offers a fast, high-resolution alternative for identifying the species present in diverse staphylococcal communities.

## 4. Conclusions

In the present study, a newly designed primer set, denoted as Tuf387/765, was developed for distinguishing staphylococcal communities at species level in a culture-independent way based on a comprehensive set of publicly available *tuf* gene sequences of staphylococcal species present in the European Nucleotide Archive database. The region of the *tuf* gene amplified by primer pair Tuf387/765 showed sufficient discriminatory power for adequate species-level identification of staphylococcal species, independent of their ecosystem of origin. When performing species-level identifications, stringent assignment criteria should be used to avoid misidentifications due to high sequence identities between closely related species. As the method developed is a culture-independent method capable of accurate identification of staphylococcal species, it offers a fast and high-resolution alternative to existing culture-dependent methods currently used to study staphylococcal communities, such as (GTG)_5_-fingerprinting and MALDI-TOF-MS. Whereas further validation of the method in real microbial ecosystems is needed, the application of the high-throughput amplicon sequencing method developed with 36 strains belonging to 18 different CNS species will allow studying complex staphylococcal communities originating from a variety of microbial environments. Such microbial ecosystems can be of both clinical and food origin, due to the widespread distribution of staphylococci across these environments.

## Figures and Tables

**Table 1:** Bacterial strains used in this study.

Species	Strain	Origin
*Staphylococcus auricularis*	IMDO-S3	Teat apex skin
	G162	Teat apex skin
*Staphylococcus arlettae*	IMDO-S2	Teat apex skin
	G238	Teat apex skin
*Staphylococcus carnosus*	IMDO-S14	Fermented meat
	IMDO-S15	Meat starter culture
*Staphylococcus chromogenes*	IMDO-S17	Teat apex skin
	IMDO-S18	Teat apex skin
*Staphylococcus cohnii*	IMDO-S19	Milk
	IMDO-S21	Teat apex skin
*Staphylococcus epidermidis*	IMDO-S29	Teat apex skin
	IMDO-S30	Fermented meat
*Staphylococcus equorum*	IMDO-S35	Fermented meat
	IMDO-S36	Fermented meat
*Staphylococcus fleuretti*	IMDO-S47	Milk
	2.05	Milk
*Staphylococcus hominis*	IMDO-S53	Unknown
	E326	Unknown
*Staphylococcus haemolyticus*	IMDO-S50	Teat apex skin
	IMDO-S51	Fermented meat
*Staphylococcus lugdunensis*	NA 14.37.1.5	Fermented meat
	IMDO-S92	Fermented meat
*Staphylococcus pasteuri*	IMDO-S54	Fermented meat
	IMDO-S55	Fermented meat
*Staphylococcus saprophyticus*	IMDO-S58	Milk
	IMDO-S59	Fermented meat
*Staphylococcus sciuri*	IMDO-S71	Teat apex skin
	IMDO-S72	Fermented meat
*Staphylococcus simulans*	IMDO-S66	Unknown
	IMDO-S95	Fermented meat
*Staphylococcus succinus*	IMDO-S67	Fermented meat
	IMDO-S68	Fermented meat
*Staphylococcus warneri*	IMDO-S73	Milk
	IMDO-S74	Milk
*Staphylococcus xylosus*	IMDO-S76	Meat starter culture
	IMDO-S81	Fermented meat

**Table 2:** Primer sets designed and used in this study. The numbers correspond to the position of the first nucleotide of the primer in the complete *tuf* gene.

Primer	Primer Sequence	Amplicon Size (bp)
Tuf108	5′- WCACGTTGACCAYGGTAAAAC -3′	301
Tuf408	5′-YTCACGMGTTTGWGGCATTGG -3′	
Tuf387	5′- YCCAATGCCWCAAACKCGTGA -3′	379
Tuf765	5′- RAYTTGHCCACGTTCAACAC -3′	
Tuf216	5′- WGAAGAAAMAGARCGTGGTA -3′	307
Tuf522	5′- RTCACGWACTTCCATTTCWACT -3′	
Tuf32	5′- AGAATAGGAGAGATTTAATAATGGC -3′	869
Tuf900	5′- VCCACGTAATAAHGCACC -3′	

## Supplementary Materials

Supplementary materials can be found at https://www.mdpi.com/2076-2607/8/6/897/s1.
