# Supplementary material for: Amplicon-Based High-Throughput Sequencing Method Capable of Species-Level Identification of Coagulase-Negative Staphylococci in Diverse Communities
Source: Microorganisms. 2020 Jun 14;8(6):897. doi: 10.3390/microorganisms8060897 (PMC7356217; doi:10.3390/microorganisms8060897)
Supplement: Supplementary file 1 [file microorganisms-08-00897-s001.pdf]

Table S1. Consensus sequence of the *tuf* gene for *Staphylococcus* spp.

atgagagataaatttgaaataacttttaytaagaataggagagatttaataatggcwaaagaaaaattygaycgytcwwwarmmcatgcc
aatatyggdacwatyggwcacgttgaccayggtaaaacdacwttaamagcdgcwatygchacwgtwwtrkcdaarmrttcyggtgay
dsdvwwgcwcrwtcwtaygmymwratyrayarygcdccwgaagaaamagarcgtggtatyacwatyaabacdkcwcayatygart
ayvmwacwrvnwmwcgtcactaygcdcaygtwgaytgyccwggwcaygckgactaygtkaadaayatgatyachggkkcdgcnca
aatggaygghgskathttrgtwgtwtcwgcwgchgayggyccaatgccwcaaackcgtgarcAYATYYTDTTATCWCGTAAYGT
WGGWGTDCCNGCDYTWGTWGTATTYTTAAAYAAAGYTGAYMWRGTWGACGAYGAAGAAYTAYTAGAATT
AGTWGAAATGGAAGTWCGTGAYYTAYTAWSHGAATAYGAYTWYCCWGGHGAYGAYRYNCCWRTWATYKY
TGGWTCWGCDYTDAAAGCDYTWGAARGCGWHVMHVVHYWHGARVVHAAAATHNTDGAMYTAATGVAH
BHDGTWGAYDMNTWYATYCCAACWCCRGAMCGYGAYTSWGAYAARCMATTCRTGATGCCDRTWGARGAY
GTWTTCTCAATHACWGGTCGYGGTACWGTWGYDACDGGBCGTGTTGAACGTGGDCAARTYAMWGTYGGY
GMHGARRTHGAAATYATYGGWHTNNMHGANVrdwBHNNNAARACWACDGTWACWGGYGTWGARATGT
TYCGTAARTTRYTRGAYTWYGCTGAAGCTGGHGAYAAYATYGGTGCDTTATTACGTGGBRTHKCDCGTGAHGA
HRTHHMHCGTGGWCAAGTWHTAGCDRMNCCHGGHWSWATYAcdccdcayacwrrvttyamwkcdgahgtwtayg
twttatcwaaagahgaaggygghcgtcwyacdccdttcttcdswaaytaycgyccrcarttytayttccgtacwacwgaygtaacwggyg
twrthmvhytacmagaagghryhgaaatggtwatgcctggbgayaayrthraaatrvnhgtwgarytrathkcwmmwatcgcdatyg
aagayggwacdcgbttytcwatycgygarggyggmcgtacwgtwggwtcaggygttgtwwcwnhhakhnwnvmngcttaa

Table S2. *In silico* analysis of *tuf* gene fragments. *S.*, *Staphylococcus*.

Species grown	Tuf32/900			Tuf108/408			Tuf387/765			Tuf216/522		
	**Proposed species**	**Sequence identity (%)**	**Accession number**	**Proposed species**	**Sequence identity (%)**	**Accession number**	**Proposed species**	**Sequence identity (%)**	**Accession number**	**Proposed species**	**Sequence identity (%)**	**Accession number**
S. arlettae	*S. saprophyticus*	94	CP022093.2	sequence could not be retrieved			*S. arlettae*	100	EU652781.1	*S. kloosi*	96	CP027846.1
S. auricularis	*S. auricularis*	99	LS483491.1	*S. auricularis*	100	LS483491.1	*S. auricularis*	100	LS483491.1	*S. auricularis*	100	LS483491.1
S. carnosus	*S. carnosus*	100	AM295250.1	*S. condementi*	100	CP018776.1	*S. carnosus*	100	CP016760.1	*S. kloosi*	96	CP027846.1
S. chromogenes	*S. hyicus*	97	LS483304.1	*S. hyicus*	97	LS483304.1	*S. chromogenes*	100	EU652790.1	*S. hyicus*	97	LS483304.1
S. cohnii	*S. cohnii*	100	CP033735.1	*S. cohnii*	100	CP033735.1	*S. cohnii*	100	CP033735.1	*S. cohnii*	100	CP033735.1
S. epidermidis	*S. epidermidis*	99	CP034115.1	*S. epidermidis*	100	CP034115.1	*S. epidermidis*	100	CP034115.1	*S. epidermidis*	100	CP034115.1
S. equorum	*S. equorum*	100	CP013980.1	*S. equorum*	100	CP013980.1	*S. equorum*	100	CP013980.1	*S. equorum*	100	CP013980.1
S. fleuretti	*S. sciuri*	97	CP020377.1	*S. sciuri*	98	CP020377.1	*S. fleuretti*	100	EU652798.1	*S. sciuri*	98	CP020377.1
S. haemolyticus	*S. haemolyticus*	99	LT963441.1	*S. hominis*	98	CP020618.1	*S. hominis*	98	CP014107.1	*S. hominis*	98	CP020618.1
S. hominis	*S. hominis*	100	LT963442.1	*S. hominis*	100	LT963442.1	*S. hominis*	100	CP033732.1	*S. hominis*	100	LT963442.1
S. lugdunensis	*S. lugdunensis*	100	CP020769.1	*S. lugdunensis*	100	LS483312.1	*S. lugdunensis*	100	LS483482.1	*S. lugdunensis*	100	LS483312.1
S. pasteuri	*S. pasteuri*	100	CP017463.1	*S. pasteuri*	100	CP017463.1	*S. pasteuri*	100	CP017463.1	*S. pasteuri*	100	CP017463.1
S. saprophyticus	*S. saprophyticus*	100	CP022093.2	*S. saprophyticus*	100	CP022093.2	*S. saprophyticus*	100	LT963436.1	*S. saprophyticus*	100	CP022093.2
S. sciuri	*S. sciuri*	99	CP020377.1	*S. sciuri*	100	CP020377.1	*S. sciuri*	100	CP020377.1	*S. sciuri*	99	CP020377.1
S. simulans	*S. simulans*	99	CP015642.1	*S. simulans*	99	CP015642.1	*S. simulans*	100	CP015642.1	*S. simulans*	100	CP015642.1
S. succinus	*S. succinus*	100	CP018199.1	*S. succinus*	100	CP018199.1	*S. succinus*	100	CP018199.1	*S. succinus*	100	CP018199.1
S. warneri	*S. warneri*	99	CP003668.1	*S. warneri*	100	CP003668.1	*S. warneri*	100	CP003668.1	*S. warneri*	100	CP003668.1
S. xylosus	*S. xylosus*	100	CP008724.1	Sequence could not be retrieved			*S. xylosus*	100	CP008724.1	*S. xylosus*	100	CP008724.1

Table S3 . Estimates of the evolutionary divergence over sequence pairs between *Staphylococcus* species. The number of base differences per sequence, averaged over all sequence pairs, between all species present in a custom *tuf* gene database are shown.

	Species	1	2	3	4	5	6	7	8	9	10	11	12	13	14	15	16	17	18	19	20	21	22	23	24	25	26	27	28	29	30	31	32	33	34	35	36	37	38	39	40	41	42	43	44	45	46	47	48
1	*Staphylococcus agnetis*																																																
2	*Staphylococcus argensis*	108																																															
3	*Staphylococcus argenteus*	88	105																																														
4	*Staphylococcus arlettae*	110	98	100																																													
5	*Staphylococcus aureus*	90	103	11	96																																												
6	*Staphylococcus auricularis*	116	80	106	84	103																																											
7	*Staphylococcus capitis*	92	83	63	71	65	81																																										
8	*Staphylococcus caprae*	93	81	57	78	59	86	12																																									
9	*Staphylococcus carnosus*	110	97	104	82	99	95	73	72																																								
10	*Staphylococcus chromogenes*	35	102	86	103	84	113	78	77	96																																							
11	*Staphylococcus cohnii*	113	108	107	73	106	88	78	80	93	103																																						
12	*Staphylococcus condimenti*	107	94	106	82	101	92	74	75	5	94	92																																					
13	*Staphylococcus delphini*	68	104	87	105	85	107	80	83	102	60	106	99																																				
14	*Staphylococcus devriesei*	99	97	64	89	65	94	35	35	84	87	101	83	92																																			
15	*Staphylococcus edaphicus*	116	108	96	66	95	91	77	76	85	109	47	84	109	94																																		
16	*Staphylococcus epidermidis*	109	80	74	75	75	74	30	34	76	91	84	77	91	47	82																																	
17	*Staphylococcus equorum*	145	134	132	120	131	127	121	127	123	136	106	122	146	134	106	124																																
18	*Staphylococcus felis*	65	96	79	100	78	100	76	77	97	71	99	94	67	79	102	86	139																															
19	*Staphylococcus fleurettii*	118	121	117	115	118	138	105	106	127	115	130	126	109	110	122	118	132	109																														
20	*Staphylococcus gallinarum*	112	97	97	75	94	92	73	71	85	102	61	83	103	89	48	77	116	93	120																													
21	*Staphylococcus haemolyticus*	97	91	55	91	57	96	37	30	82	84	95	82	88	27	84	54	131	78	108	85																												
22	*Staphylococcus hominis*	105	103	66	90	70	89	46	42	82	92	97	82	88	38	88	59	131	87	111	89	26																											
23	*Staphylococcus hyicus*	19	111	89	105	90	122	95	96	107	38	107	104	70	98	106	107	142	72	118	105	94	103																										
24	*Staphylococcus intermedius*	62	102	78	100	81	107	76	78	99	61	100	96	14	87	99	85	141	56	105	97	82	82	65																									
25	*Staphylococcus kloosii*	103	83	98	59	100	74	72	76	80	96	68	77	99	94	85	74	100	99	118	80	93	90	107	94																								
26	*Staphylococcus lentus*	122	134	123	125	130	144	114	114	138	119	143	137	119	124	133	127	145	121	49	129	122	120	123	116	124																							
27	*Staphylococcus lugdunensis*	105	95	86	93	84	98	66	60	79	91	98	80	98	64	96	71	126	92	111	97	57	60	98	96	93	121																						
28	*Staphylococcus lutrae*	97	122	110	122	107	122	105	108	121	93	114	118	58	112	121	117	152	80	120	115	109	106	97	55	107	129	116																					
29	*Staphylococcus massiliensis*	106	79	98	97	98	84	82	78	100	105	104	99	94	95	101	88	136	92	125	97	91	94	107	87	87	131	93	115																				
30	*Staphylococcus microti*	76	108	96	96	94	94	72	78	88	73	104	85	67	73	94	80	132	72	111	88	80	79	80	59	95	115	95	87	99																			
31	*Staphylococcus muscae*	76	109	96	97	96	101	79	87	92	75	102	89	74	79	95	87	125	77	108	91	82	84	78	65	98	112	101	92	107	22																		
32	*Staphylococcus nepalensis*	122	110	103	74	103	91	78	76	91	108	37	90	113	98	56	86	107	106	128	67	92	92	115	107	70	138	94	129	103	109	112																	
33	*Staphylococcus pasteuri*	99	86	72	93	76	91	45	44	88	90	95	88	92	51	89	57	136	88	110	92	50	58	95	89	86	122	67	112	96	82	92	94																
34	*Staphylococcus petrasii*	96	97	63	91	65	94	39	40	87	88	106	87	93	27	97	55	129	86	113	95	22	34	97	86	93	128	64	116	91	82	84	104	58															
35	*Staphylococcus pettenkoferi*	106	8	103	99	100	78	83	81	99	102	107	94	102	97	108	82	134	94	123	94	91	105	110	100	82	135	99	120	77	104	105	111	86	97														
36	*Staphylococcus pseudintermedius*	69	102	85	104	83	104	78	81	99	59	103	96	7	90	105	88	143	65	108	100	86	86	71	15	98	117	96	54	91	65	74	109	91	93	100													
37	*Staphylococcus rostri*	77	106	93	96	91	97	76	82	88	69	102	85	66	76	97	84	130	74	107	89	77	79	75	62	95	115	89	90	102	12	16	109	83	81	102	66												
38	*Staphylococcus saprophyticus*	114	105	100	69	99	90	78	77	84	105	52	83	109	96	14	83	108	101	118	42	87	90	107	101	82	127	97	121	97	94	97	56	88	99	104	104	97											
39	*Staphylococcus schleiferi*	75	102	86	93	88	98	75	78	92	62	93	89	54	82	98	80	131	67	114	98	79	81	75	53	82	123	95	71	92	65	70	100	87	85	101	50	65	99										
40	*Staphylococcus schweitzeri*	89	107	8	98	18	105	64	61	106	88	106	108	86	68	97	76	131	79	115	97	61	69	90	77	98	121	88	109	100	95	93	100	79	69	106	85	92	100	85									
41	*Staphylococcus sciuri*	109	115	114	112	116	133	103	101	127	109	130	126	108	105	124	115	135	103	33	121	101	107	105	101	114	50	103	113	116	99	99	125	104	108	116	108	96	120	107	111								
42	*Staphylococcus simiae*	95	99	48	83	51	93	57	59	89	88	94	91	95	65	90	72	122	85	116	93	63	69	100	86	78	123	81	112	94	98	95	88	74	63	101	92	96	91	87	48	110							
43	*Staphylococcus simulans*	103	99	104	92	100	94	76	77	30	99	103	30	106	83	94	80	134	95	128	89	85	85	102	101	85	140	91	124	100	93	98	104	83	89	96	103	95	90	92	108	130	87						
44	*Staphylococcus stepanovicii*	116	126	135	120	134	132	124	123	132	120	142	131	119	130	135	134	146	116	54	131	124	127	122	116	124	63	123	125	134	112	112	137	115	132	126	117	110	126	119	133	48	131	131					
45	*Staphylococcus succinus*	124	112	104	85	102	101	90	87	94	110	67	93	110	110	55	91	108	116	116	63	101	97	117	107	97	124	100	126	112	104	100	68	105	111	111	107	101	54	108	103	123	95	100	128				
46	*Staphylococcus vitulinus*	123	137	133	126	136	149	118	118	135	120	146	134	125	121	140	130	147	122	41	142	119	122	124	120	129	51	116	131	138	120	118	143	121	126	139	124	120	135	124	131	46	128	137	54	137			
47	*Staphylococcus warneri*	92	91	66	90	71	89	41	41	87	79	92	87	86	51	90	58	136	84	116	85	49	56	91	82	86	127	73	102	88	76	88	94	28	56	90	82	80	90	73	69	111	71	77	128	104	124		
48	*Staphylococcus xylosus*	120	116	109	74	108	98	95	96	99	116	60	98	120	110	39	100	100	114	132	59	103	103	117	111	80	137	106	128	104	111	108	61	112	112	119	116	111	37	114	108	131	98	110	144	68	144	109	

Table S4. Estimates of the average evolutionary divergence over sequence pairs within *Staphylococcus* species. Cases for which it was not possible to estimate evolutionary distances, due to the presence of a single sequence in the database, are denoted with na (non-applicable).

**Species**	**Number of nucleotide differences**
Staphylococcus agnetis	1
Staphylococcus argensis	na
Staphylococcus argenteus	1
Staphylococcus arlettae	0
Staphylococcus aureus	3
Staphylococcus auricularis	4
Staphylococcus capitis	0
Staphylococcus caprae	0
Staphylococcus carnosus	0
Staphylococcus chromogenes	3
Staphylococcus cohnii	14
Staphylococcus condimenti	0
Staphylococcus delphini	0
Staphylococcus devriesei	3
Staphylococcus edaphicus	na
Staphylococcus epidermidis	1
Staphylococcus equorum	4
Staphylococcus felis	2
Staphylococcus fleurettii	0
Staphylococcus gallinarum	6
Staphylococcus haemolyticus	3
Staphylococcus hominis	6
Staphylococcus hyicus	1
Staphylococcus intermedius	na
Staphylococcus kloosii	0
Staphylococcus lentus	4
Staphylococcus lugdunensis	2
Staphylococcus lutrae	na
Staphylococcus massiliensis	na
Staphylococcus microti	0
Staphylococcus muscae	na
Staphylococcus nepalensis	1
Staphylococcus pasteuri	0
Staphylococcus petrasii	11
Staphylococcus pettenkoferi	3
Staphylococcus pseudintermedius	1
Staphylococcus rostri	na
Staphylococcus saprophyticus	7
Staphylococcus schleiferi	1
Staphylococcus schweitzeri	na

Staphylococcus sciuri	5
Staphylococcus simiae	na
Staphylococcus simulans	1
Staphylococcus stepanovicii	na
Staphylococcus succinus	4
Staphylococcus vitulinus	1
Staphylococcus warneri	3
Staphylococcus xylosus	11

Table S5. Comparison of expected and observed relative abundance of staphylococcal species for mock communities M1, M2, and M3, and amplicon-based mock communities R1, R2, R3, R4, and R5 based on amplicon sequencing results with primer pair Tuf387/765

Species	R1		R2		R3		R4		R5		M1		M2		M3	
	Expected abundance (%)	Observed abundance (%)	Expected abundance (%)	Observed abundance (%)	Expected abundance (%)	Observed abundance (%)	Expected abundance (%)	Observed abundance (%)	Expected abundance (%)	Observed abundance (%)	Expected abundance (%)	Observed abundance (%)	Expected abundance (%)	Observed abundance (%)	Expected abundance (%)	Observed abundance (%)
Staphylococcus carnosus	9,53	9,89	10,66	11,50	8,48	9,28	9,12	11,72	8,01	10,96	12,91	25,84	0,05	0,06	32,74	35,83
Staphylococcus epidermidis	12,30	12,62	13,76	12,38	10,94	10,76	11,78	11,36	10,34	9,95	10,25	6,94	0,02	0,03	0,04	0,02
Staphylococcus equorum	8,44	10,38	9,43	8,34	7,50	7,70	8,07	9,50	7,09	8,60	10,25	6,51	13,34	9,11	0,26	0,05
Staphylococcus haemolyticus	0.00	0.00	0.00	0.00	11,08	9,86	0.00	0.00	0.00	0.00	0.00	0.00	0.00	0.00	0.00	0.00
Staphylococcus hominis	10,55	6,41	0.00	0.00	9,38	6,78	0.00	0.00	0.00	0.00	0.00	0.00	0.00	0.00	0.00	0.00
Staphylococcus lugdunensis	15,04	14,03	16,81	17,21	13,37	13,12	14,39	15,20	12,64	13,22	0.00	0.00	0.00	0.00	0.00	0.00
Staphylococcus pasteuri	0.00	0.00	0.00	0.00	0.00	0.00	0.00	0.00	12,21	8,72	0.00	0.00	0.00	0.00	0.00	0.00
Staphylococcus saprophyticus	10,47	8,04	11,70	8,27	9,31	7,27	10,02	7,78	8,80	7,21	40,82	16,68	53,09	19,75	1,64	0,27
Staphylococcus sciuri	6,41	7,91	7,16	9,22	5,70	6,86	6,13	7,33	5,38	6,36	0.00	0.00	0.00	0.00	0.00	0.00
Staphylococcus succinus	14,26	11,13	15,94	11,64	12,68	9,40	13,64	10,02	11,98	9,35	0.00	0.00	0.00	0.00	0.00	0.00
Staphylococcus warneri	0.00	0.00	0.00	0.00	0.00	0.00	14,39	7,65	12,64	7,51	0.00	0.00	0.00	0.00	0.00	0.00
Staphylococcus xylosus	13,01	11,80	14,54	12,09	11,57	10,68	12,45	11,43	10,93	11,17	25,76	28,80	33,50	43,26	65,32	62,72
